# SVRMHC prediction server for MHC-binding peptides

**DOI:** 10.1186/1471-2105-7-463

**Published:** 2006-10-23

**Authors:** Ji Wan, Wen Liu, Qiqi Xu, Yongliang Ren, Darren R Flower, Tongbin Li

**Affiliations:** 1 Department of Neuroscience, University of Minnesota, Minneapolis, MN 55455, USA; 2 The Jenner Institute, University of Oxford, Compton, Berkshire RG20 7NN, UK

## Abstract

**Background:**

The binding between antigenic peptides (epitopes) and the MHC molecule is a key step in the cellular immune response. Accurate *in silico *prediction of epitope-MHC binding affinity can greatly expedite epitope screening by reducing costs and experimental effort.

**Results:**

Recently, we demonstrated the appealing performance of SVRMHC, an SVR-based quantitative modeling method for peptide-MHC interactions, when applied to three mouse class I MHC molecules. Subsequently, we have greatly extended the construction of SVRMHC models and have established such models for more than 40 class I and class II MHC molecules. Here we present the SVRMHC web server for predicting peptide-MHC binding affinities using these models. Benchmarked percentile scores are provided for all predictions. The larger number of SVRMHC models available allowed for an updated evaluation of the performance of the SVRMHC method compared to other well- known linear modeling methods.

**Conclusion:**

SVRMHC is an accurate and easy-to-use prediction server for epitope-MHC binding with significant coverage of MHC molecules. We believe it will prove to be a valuable resource for T cell epitope researchers.

## Background

Major histocompatibility complex molecules (MHCs) are polymorphic glycoproteins residing on cell membranes. In the cellular immune system, MHC molecules bind small peptide fragments, or epitopes, derived from antigens and host proteins, and present them to T cells, thus inducing downstream immune system responses. Computational prediction and modeling of epitope-MHC binding is of considerable interest because it can greatly facilitate epitope screening, with tremendous concomitant savings in time and experimental effort. Over the past ~15 years, many such computational methods have been proposed (for a comprehensive review see [1]). While some of these methods are structure-based (e.g., [2-5]) or make use of structural information (e.g., [6]), the majority of methods are sequence-based. While interesting and bursting with potential, structure- based methods are currently less reliable than strongly data-driven sequence-based methods. In terms of the types of predictions made, sequence-based methods are of two types. Most methods, including BIMAS [7], SYFPEITHI [8], RANKPEP [9], SVMHC [10], MULTIPRED [11], and a few others, e.g., [12-14] are "qualitative methods", i.e., they make predictions about whether a peptide is a "binder" or a "non-binder" or a "strong binder" or a "weak binder". Some recent methods, including 3D-QSAR [15] and the additive method [16,17], are "quantitative" data-driven techniques, i.e., they predict the exact binding affinity of the peptide.

We recently developed SVRMHC, a support vector machine regression (SVR)-based method for modeling peptide-MHC binding. SVRMHC is a sequence-based quantitative method that makes predictions about the exact binding affinity of the peptide. As a kernel-based approach, SVRMHC demonstrates the excellent modeling performance enjoyed by other SVM-based methods such as SVMHC [10] and HLA-DR4Pred [18]. In a preliminary test with three mouse class I MHC alleles (H2-Db, H2-Kb and H2-Kk), we showed that SVRMHC produced models that out-performed those generated using the linear additive method. Moreover, a Receiver Operating Characteristic (ROC)-based comparison suggested that SVRMHC out-performed prominent methods in identifying strongly binding peptides [19].

Subsequently, we constructed and validated SVRMHC models for over 40 MHC alleles. In this report, we describe the SVRMHC server, which predicts T-cell epitopes using these models. In addition to the predicted binding affinity, the SVRMHC server calculates a percentile score for each input peptide benchmarked against a pool of ~528,500 peptides. These were derived from 1,000 proteins picked randomly from the Swiss-Prot database. Construction of a large number of SVRMHC models has allowed a better comparison to be made between the SVRMHC and the additive method, which we discuss briefly in this report.

## Implementation

SVRMHC model construction was carried out in locally developed C and Matlab programs. LibSVM was used for SVR-related implementation [20]. The web server was developed as a PHP project running under Apache 2.0 on a Fedora Core II Linux system.

## Results

### Construction of SVRMHC models

The data used for constructing the SVRMHC models was obtained from the AntiJen database [21] (March 3, 2006). Each binding experiment was represented as a (sequence:pIC50) pair in the dataset. We constructed SVRMHC models for all class I MHC alleles with ≥ 30 affinity measurements and all class II alleles with ≥ 50 affinity measurements. In total, models for 42 MHC molecules (36 class I, 6 class II) were constructed (Tables 1 and 2). They included 37 human, 3 mouse, and 2 chimpanzee MHC molecules. For each MHC molecule, we attempted six different configurations resulting from three different kernel functions (linear, polynomial and RBF) in combination with two sequence encoding schemes ("sparse encoding", and "11-factor encoding" [19]). The accuracy of prediction for each configuration was assessed using cross-validated *q*^*2 *^(for class I models) or cross-validated *r *(for class II models). The configuration that offers the highest prediction performance was chosen for the final model. LOO (leave-one-out) or 7-fold cross-validation was used when assessing the performance of class I models, and 5-fold cross-validation was used when evaluating class II model performance. The final model set included 39 nonamer models, together with 2 octamer models (for H2-Kb and H2-Kk) and 1 decamer model (for A*0207).

**Table 1:** The list of class I MHC alleles for which SVRMHC models have been constructed.

**MHC allele**	**Linear, 11-factor**	**Linear, Sparse**	**Polynomial, 11-factor**	**Polynomial, Sparse**	**RBF, 11-factor**	**RBF, Sparse**
A*0101	0.228	0.172	0.237	**0.353**	0.339	0.344
A*0201	0.245	0.211	0.383	0.433	**0.485**	0.461
A*0202	-0.173	-0.709	0.115	**0.273**	0.205	0.228
A*0203	0.189	-0.009	**0.352**	0.291	0.346	0.297
A*0204	-0.695	-0.691	0.007	-0.01	**0.031**	-0.02
A*0206	0.066	0.325	0.266	0.369	0.272	**0.38**
A*0207	0.682	0.619	**0.682**	0.629	0.68	0.628
A*0301	0.204	0.284	0.361	0.431	**0.534**	0.374
A*0302	-0.057	0.189	0.174	**0.208**	0.172	0.207
A1	0.25	0.31	0.26	**0.382**	0.36	0.379
A11	0.1	-0.546	0.334	0.263	**0.336**	0.279
A*1101	0.09	-0.118	0.197	0.202	**0.206**	0.197
A2	0.158	0.109	0.315	0.304	**0.342**	0.316
A24	0.205	0.1	0.361	0.21	**0.378**	0.233
A3	0.023	-0.361	0.293	0.348	**0.373**	0.357
A31	-0.038	0.268	0.217	0.392	**0.395**	0.389
A*3101	0.743	0.385	**0.743**	0.487	0.741	0.492
A33	-0.777	0.079	0.004	**0.245**	0.16	0.224
A*3301	-0.777	0.079	0.004	**0.245**	0.16	0.224
A68	0.278	0.223	0.332	0.398	0.347	**0.421**
A*6801	0.00014	0.287	**0.408**	0.293	0.394	0.312
A*6802	-0.169	0.201	0.001	0.313	0.243	**0.344**
B*0702	0.19	0.221	0.349	0.398	**0.422**	0.413
B35	-0.132	0.333	0.171	0.363	**0.382**	0.36
B*3501	-0.397	0.113	0.193	**0.26**	0.24	0.26
B51	0.492	0.145	0.424	0.408	**0.507**	0.408
B53	0.073	0.508	0.25	0.445	0.289	**0.507**
B*5301	0.073	0.508	0.25	**0.508**	0.289	0.507
B54	0.468	-0.212	**0.468**	0.269	0.429	0.277
B*5401	0.468	-0.212	**0.468**	0.269	0.429	0.277
B7	0.343	0.223	0.328	0.528	0.443	**0.543**
H-2Db	0.504	-0.038	**0.552**	0.412	0.521	0.416
H-2Kb	-0.09	-0.526	0.259	0.18	**0.28**	0.178
H-2Kk	0.731	0.501	0.738	0.502	**0.763**	0.513
Mamu-B*17	0.621	0.595	0.554	0.64	0.602	**0.653**
Patr-A*0602	-0.143	0.412	0.318	0.447	0.171	**0.476**

The table also contains statistics for the performance of the models (expressed in cross-validated *q*^*2*^) for various configurations of parameters. The configurations offering the best performance are marked in bold, and these are the models implemented in the SVRMHC server.

**Table 2:** The list of class II MHC alleles for which SVRMHC models have been constructed.

**MHC allele**	**Linear, 11-factor**	**Linear, Sparse**	**Polynomial, 11-factor**	**Polynomial, Sparse**	**RBF, 11-factor**	**RBF, Sparse**
DRB1*0401	0.526	0.556	0.551	**0.612**	0.582	0.61
DRB1*0101	0.531	0.5	0.568	0.616	**0.634**	0.61
DRB1*1501	0.659	0.622	0.703	0.693	**0.7078**	0.671
DQA1*0501	0.456	0.568	0.529	**0.581**	0.546	0.537
DRB1*0405	0.249	**0.48**	0.364	0.415	0.295	0.412
DRB5*0101	0.408	0.479	0.391	**0.589**	0.374	0.532

The table also includes statistics of performance for the models (expressed in cross-validated *r*) for various configurations of parameters. The configurations offering the best performance are marked in bold, and these are the models implemented in the SVRMHC server.

The class II SVRMHC model construction was more complicated than the class I case because the longer input sequences required alignment to the model's nonameric "core sequence". We took an approach similar to the iterative self-consistent (ISC) strategy described earlier [17]. First, we obtained the anchor position information about the class II MHC molecule from SYFPEITHI [8]. The first anchor position was used to limit the number of possible alignments to be considered: only alignments with a reported anchor amino acid at the first anchor position were considered to be valid. At the beginning of model construction, all validly aligned nonamer sequences, as derived from all training set sequences, were included in the model training. After the first model was trained, predictions were made for each aligned sequence. The alignment for each input sequence that resulted in the smallest residual in the prediction was retained, and other alternative alignments were removed. A subsequent model was then trained using the updated set of aligned sequences; after this, another round of predictions was made. This process continued until the model performance (as measured by cross-validated *r*) no longer improved, or when an iteration threshold was exceeded (this number was set to 4).

Three different sequence alignment protocols – "mean", "max", and "combi" – were used in [17] when making predictions for a sequence with an established model. Our present experience with the SVRMHC models indicated that no significant difference was apparent among the three alignment protocols. However, overall the "mean" alignment method offered slightly better cross-validated *r *scores. Therefore, "mean" alignment was implemented in the SVRMHC server.

### Benchmarking prediction results

In ROC-based comparisons, previous SVRMHC models out-performed several well-known methods when identifying strong binding peptides [19]. This suggests that SVRMHC models perform well in sorting peptides in terms of their relative binding affinities. However, the absolute values of predictions made by SVRMHC models may be sensitive to bias introduced into the dataset used to train the models. For instance, if the training dataset mainly consists of strong binders (pIC50>7), then the constructed model is likely to be biased towards a higher affinity predictions range. To counter this potential problem, we benchmarked each SVRMHC model using a large number of natural peptide sequences. We picked 800 human proteins and 200 mouse proteins at random from the Swiss-Prot database. From these 1000 proteins, we extracted all short subsequences of length 8, 9, and 10. After removal of identical sequences, 528,409 octamers, 528,596 nonamers and 528,433 decamers were obtained. These sequences constituted the benchmark sequence pool. For each SVRMHC model, predictions were made using all sequences in this pool, and the distribution of predicted values was obtained. This distribution provides an estimate of how the "general population" of peptides would "behave" when calculated using the SVRMHC model. The higher the rank of a peptide relative to the "general population", the more likely it is to be a strong binder. Likewise, a low ranked peptide may not be a stronger binder even if its predicted binding value is high (e.g. pIC50>7). Thus, for each peptide sequence submitted by the user, the SVRMHC server provides not only the predicted binding affinity of the peptide, but also a percentile score revealing how many sequences in the benchmark pool produced higher predicted binding affinity values than the sequence of interest.

### Utility

At the SVRMHC prediction server, the user can paste a protein sequence (either as plain text or in FASTA format) into the "Input Sequence" text area, or upload a local sequence file to the server. The user then selects the target MHC allele. Optionally, the user can enter either a pIC50 threshold or a percentile score threshold. The prediction results (pIC50 values and percentile scores) will be displayed either in the order in which they occur in the input protein sequence or sorted as a list in descending order of predicted pIC50 values.

## Discussion

### Model configuration statistics

Of the 42 final SVRMHC models included in the server (see Tables 1 and 2), 23 were constructed using the RBF kernel, 18 were constructed using the polynomial kernel, and one was constructed using the linear kernel. In 23 out of the 42 final models, the "11-factor encoding" scheme was adopted; the remaining 19 final models used the "sparse encoding" scheme. The number of final models that adopted the four configurations "RBF/11-factor", "RBF/sparse", "polynomial/11-factor", and "polynomial/sparse" were 16, 7, 7 and 11, respectively. These statistics suggest that although the configuration "RBF/11-factor" is most likely to generate the best performing model, it is possible for other configurations to produce better models. It is therefore sensible, given a new dataset, to explore all configurations and identify that which offers optimal performance.

### Performance comparison with linear modeling methods

In our previous report [19], we showed that SVRMHC models offered better performance than models constructed using the linear "additive method" using binding datasets for three mouse class I MHC alleles. Having constructed larger numbers of models, we could now compare the two approaches more completely. We built "additive method" models for the 42 MHC molecules as described in [16,17], with the same datasets used to construct corresponding SVRMHC models. A comparison between the SVRMHC models and the "additive method" models indicated that the SVRMHC models produced significantly higher cross-validated *q*^*2 *^than the "additive method" models before outlier removal [19,22]. However, after we removed outliers, the performance of SVRMHC and "additive method" models was comparable, though fewer outliers were removed for the SVRMHC models. More details of the comparisons can be found at [23].

## Conclusion

SVRMHC server is an accurate and easy-to-use server for predicting epitope-MHC binding. It offers significant coverage in terms of MHC molecules and this study has reconfirmed model performance. SVRMHC will continue to expand as more binding data becomes available. We believe the SVRMHC server will become a valuable resource for researchers interested in predicting T cell epitopes.

## Availability and requirements

SVRMHC server is publicly accessible from the URL . Questions and comments are welcomed through the site.

## Authors' contributions

JW carried out some of the SVRMHC model construction work, most of the benchmarking and statistic analysis, and produced all compiled models for server construction. WL constructed the majority of SVRMHC models, and performed analysis on model configurations. QX organized the binding data from AntiJen, and executed most of the additive model construction work for performance comparison with SVRMHC models. YR constructed the server web site. DRF provided the data for constructing the SVRMHC models, gave significant assistance and advice on essential issues of the model construction, and helped to write the manuscript. TL conceived of and coordinated the study, performed some of the analysis, and drafted and finalized the manuscript. All authors read and approved the final manuscript.
